# Associations between lipoproteins and risk of ischemic stroke: a systematic review and meta-analysis of Mendelian randomization studies

**DOI:** 10.3389/fneur.2025.1694731

**Published:** 2026-01-06

**Authors:** Yanwei Guo, Zhijian Guo, Yinnan Zhu, Zongzhi Xie, Caixin Yang, Shouyuan Sun

**Affiliations:** 1 Lanzhou University Second Clinical Medical School, Lanzhou, Gansu, China; 2 Department of Neurosurgery, Lanzhou University Second Hospital, Lanzhou, Gansu, China; 3 The Second People’s Hospital of Gansu Province, Lanzhou, Gansu, China

**Keywords:** ischemic stroke, lipoproteins, Mendelian randomization (MR) studies, meta-analysis, systematic review

## Abstract

**Objective:**

To systematically evaluate the causal effects of lipoproteins on ischemic stroke (IS) through a systematic review and meta-analysis of Mendelian randomization (MR) studies.

**Methods:**

A comprehensive literature search was conducted in PubMed, Embase, Cochrane Library, and Web of Science to identify MR studies investigating the relationship between lipoproteins and IS, covering all publications up to November 2024. Relevant data were extracted, followed by a quality assessment. Meta-analyses were performed using RevMan software, with evaluations of heterogeneity and publication bias.

**Results:**

A total of 442 studies were evaluated, and 10 were included. Our meta-analysis showed a significant positive correlation between LDL and IS (OR 1.09, 95% CI 1.07–1.12; *p* < 0.001, *I*^2^ = 0), apoB and IS (OR 1.08, 95% CI 1.02–1.14; *p* = 0.006, *I*^2^ = 60%), VLDL and IS (OR 1.09, 95% CI 1.01–1.18; *p* = 0.03). In contrast, apoA1 was significantly negatively associated with IS (OR 0.93, 95% CI 0.89–0.96; *p* < 0.001, *I*^2^ = 38%). Interestingly, although the association between Lp(a) and IS was not statistically significant (OR 1.01, 95% CI 0.99–1.02; *p* = 0.26, *I*^2^ = 57%), combined non-HDL lipoproteins were positively associated with IS (OR 1.03, 95% CI 1.01–1.06; *p* < 0.001, *I*^2^ = 93.6%). In contrast, HDL lipoproteins was significantly negatively associated with IS (OR 0.93, 95% CI 0.89–0.97; *p* < 0.001, *I*^2^ = 58%). The funnel plot appears generally symmetrical and sensitivity analyses confirmed the robustness of the findings for LDL, apoA1, apoB, HDL, and total cholesterol in relation to IS.

**Conclusion:**

This meta-analysis provides evidence for a causal relationship between various lipoproteins and ischemic stroke. Most non-HDL lipoproteins (LDL, VLDL, apoB) are associated with an increased risk of IS, while HDL and apoA1 appear to confer a protective effect. The role of Lp(a) in IS remains inconclusive and warrants further investigation.

**Systematic review registration:**

https://www.crd.york.ac.uk/PROSPERO, CRD42024617825.

## 1 Introduction

Stroke is a condition characterized by damage to the cerebral vasculature caused by various factors, leading to focal neurological deficits. It remains one of the leading causes of death and long-term disability worldwide (1). Among all stroke cases globally, ischemic stroke (IS) accounts for approximately 62–67% (2), with large vessel atherosclerosis being a predominant underlying cause (3). Major risk factors for IS include hypertension, diabetes mellitus, smoking, high cholesterol, and obesity (4). Understanding the causal relationships between lipoproteins and IS could aid in reducing its incidence, offering important clinical implications for developing targeted prevention strategies (5).

Lipoproteins differ substantially in their proteolipid composition, and each type plays a distinct role in the pathophysiology of IS (6). These structural and functional differences make the association between lipoproteins and stroke risk complex. For example, low-density lipoprotein (LDL) is primarily responsible for transporting cholesterol to peripheral tissues and is strongly linked to atherosclerosis. Similarly, lipoprotein(a) [Lp(a)] exhibits pro-atherogenic, pro-inflammatory, pro-thrombotic, and anti-fibrinolytic properties, further implicating it in vascular pathology (7). However, a focus solely on traditional metrics like LDL cholesterol (LDL-C) and high-density lipoprotein cholesterol (HDL-C) provides an incomplete picture. Apolipoprotein B (apoB) represents the total number of atherogenic particles (including LDL, VLDL, and others) and is increasingly recognized as a potentially superior predictor of cardiovascular risk. Conversely, apolipoprotein A1 (apoA1) is the main functional component of HDL, influencing its efficiency in reverse cholesterol transport. Likewise, very-low-density lipoprotein (VLDL) serves as a key precursor in triglyceride metabolism and contributes to atherosclerosis independently. While numerous studies have suggested correlations between circulating lipoprotein levels and IS risk, the precise nature of these relationships remains ambiguous.

Mendelian randomization (MR) is a genetic epidemiological method that employs genetic variants associated with modifiable exposures—such as lipid levels—as instrumental variables (IVs) to infer causal relationships between those exposures and disease outcomes (8). Compared to traditional observational studies, MR is less susceptible to confounding because genetic variants are randomly assorted at conception. Moreover, since genetic variants are fixed before disease onset, MR helps mitigate reverse causality bias (9). Given these methodological strengths, MR has been increasingly applied to investigate the independent effects of low-density lipoprotein cholesterol (LDL-C), triglycerides, and high-density lipoprotein cholesterol (HDL-C) on IS risk (10). However, inconsistencies remain, partly due to genetic pleiotropy and other methodological limitations, particularly when examining lipoprotein subtypes (11, 12).

Despite the growing body of MR-based research, no comprehensive systematic review has yet synthesized the available evidence on the causal relationships between lipoproteins and IS. Most existing reviews and meta-analyses have focused predominantly on LDL-C, HDL-C, and triglycerides, leaving the causal evidence for apoB, apoA1, VLDL, and Lp(a) systematically underexplored and fragmented. To address this gap, we conducted a systematic review and meta-analysis of MR studies evaluating the associations between various lipoproteins and IS. This analysis aims to clarify the role of lipoproteins in stroke pathogenesis and provide more robust evidence to inform prevention and intervention strategies.

## 2 Methods

This study follows the Preferred Reporting Items for Systematic Reviews and Meta-Analyses (PRISMA) guidelines (13). Additionally, the protocol is registered with PROSPERO (CRD42024617825).

### 2.1 Search strategy

We conducted a comprehensive search of four databases—PubMed, Embase, Cochrane Library, and Web of Science—from their inception until November 22, 2024. Our search strategy included the following terms: (“Lipoproteins” OR “Circulating Lipoproteins”) AND (“Ischemic Stroke” OR “Cryptogenic Ischemic Stroke”). Additionally, we incorporated terms such as “Mendelian Randomization Analysis,” “Genome-Wide Association Study,” and “Instrumental Variables” to identify studies utilizing this specific study design. The complete search strategy for each database is detailed in Supplementary Material 1. Furthermore, we thoroughly examined the reference lists of the included studies to identify any additional relevant articles that may have been missed during the initial search, ensuring a comprehensive and exhaustive approach.

### 2.2 Inclusion and exclusion criteria

The inclusion criteria for this study were as follows: (1) studies employing MR methodology to assess the causal relationship between lipoproteins and ischemic stroke; (2) genome-wide or phenotype-wide association studies (GWAS/PheWAS) incorporating MR as part of their analysis; (3) studies reporting outcomes as 95% confidence intervals (CIs), odds ratios (ORs), and relative risks (RRs), or providing raw data convertible into these metrics.

The exclusion criteria for this study were as follows: (1) studies involving non-human subjects; (2) duplicate publications; (3) non-peer-reviewed preprints; (4) reviews, conference abstracts, letters to the editor, editorials, or case reports; and (5) studies without full-text availability.

### 2.3 Study selection

All identified literature was imported into EndNote X9 for duplicate removal. Two researchers independently screened records by title and abstract, followed by full-text assessment against predefined eligibility criteria. Inter-rater agreement during full-text screening was quantified using Cohen’s kappa, demonstrating a high degree of consistency between reviewers. Discrepancies identified during screening were primarily resolved through discussion. In the few cases where consensus was not achieved, a third independent researcher was consulted to make the final determination.

### 2.4 Quality assessment and data extraction

The following data were extracted: the first author’s name, year of publication, study design, participant characteristics (exposures and outcomes), number of instrumental variables (SNPs), data source (for exposures and outcomes), sample size (for exposures and outcomes), and MR methodology. When a single article reported multiple exposure-outcome associations, each association was treated as a separate study. If multiple articles used data from the same source or database, only the one with the largest outcome sample size was included.

The quality of the included studies was assessed based on adherence to the Strengthening the Reporting of Mendelian Randomization Studies (STROBE-MR) guidelines (14). These guidelines were adapted for studies reporting quality assessment methods used in MR research (15, 16). The assessment was based on the 21-item STROBE-MR checklist. Each item was scored equally, and a total percentage score was calculated for each study. To categorize study quality, we applied the following conventional thresholds: studies scoring below 75% were considered low quality, those scoring between 75 and 85% as moderate quality, and those scoring above 85% as high quality. This classification system is consistent with methodological standards used in systematic reviews and reflects the degree of adherence to rigorous reporting practices.

Data extraction and methodological quality assessment were conducted independently by two researchers. Any discrepancies were resolved through consultation with a third researcher.

### 2.5 Statistical analysis

Review Manager (version 5.3) was used to conduct all statistical analyses. The odds ratios (ORs) from individual studies were pooled using a random-effects model. Forest plots were generated to visually assess the combined results.

Heterogeneity among studies was evaluated using the *I*^2^ statistic, where values between 25 and 50% indicated mild heterogeneity, between 50 and 75% indicated moderate heterogeneity, and values greater than 75% indicated severe heterogeneity. Sensitivity analyses were performed using the leave-one-out method to determine the influence of each study on the overall effect size estimate. Additionally, funnel plot analysis was conducted to assess publication bias.

## 3 Results

### 3.1 Literature screening and selection

**Figure 1:**
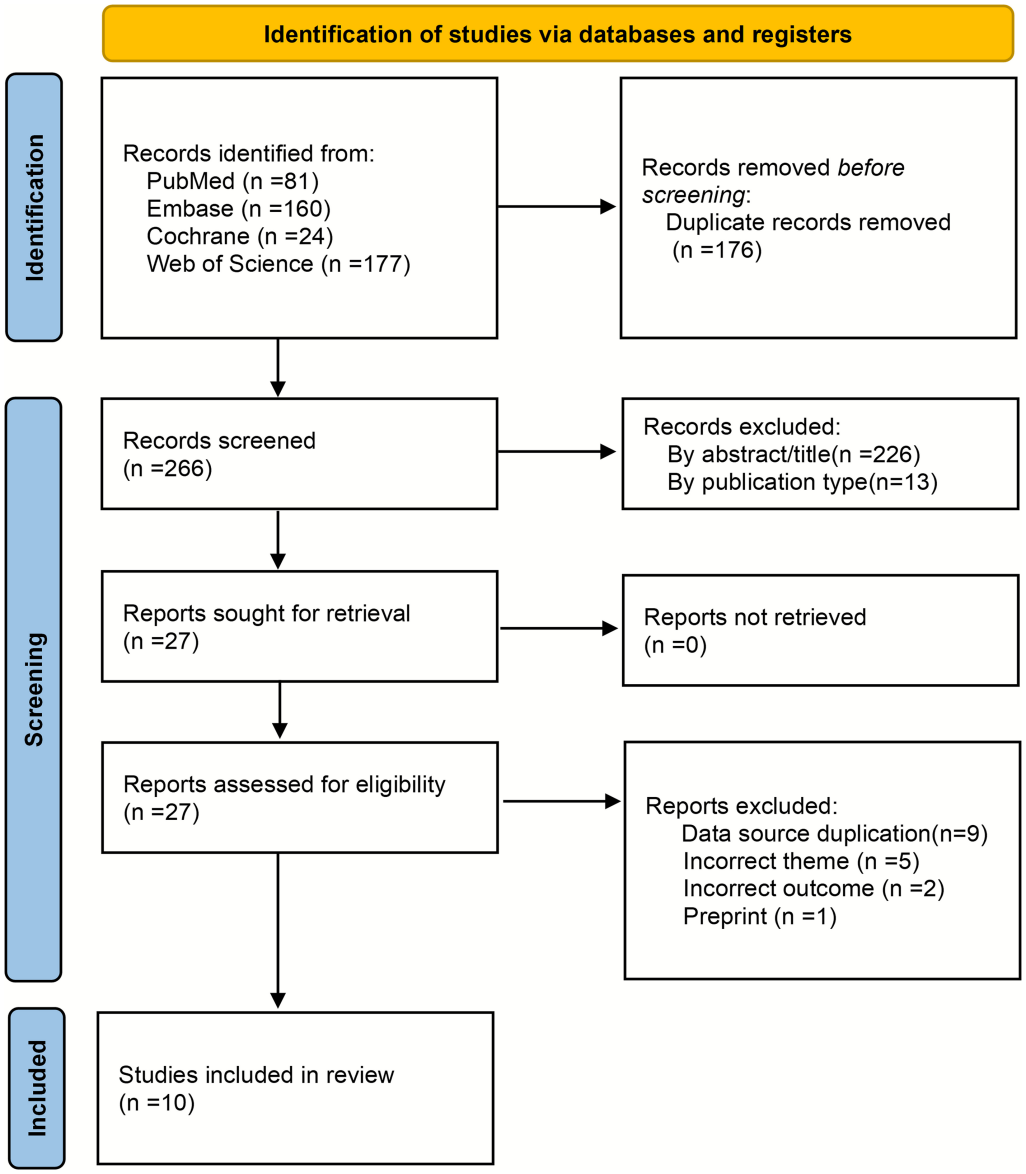
PRISMA flow diagram of the study process.

The initial search identified 442 articles from major biomedical databases, including PubMed, Embase, Cochrane Library, and Web of Science. After removing 176 duplicate articles, 266 remained for further screening. Following the application of eligibility criteria, 239 records were excluded, leaving 27 articles for full-text eligibility assessment. Of these, 17 full-text records were excluded after detailed evaluation. The primary reasons for exclusion included: study design not based on MR overlapping data sources with other included studies, and the exposure or outcome not meeting our predefined eligibility criteria. This process resulted in the inclusion of 10 studies in the final meta-analysis (11, 12, 17–24). The screening process and its results are illustrated in Figure 1.

### 3.2 Characteristics and quality of the included studies

We carefully analyzed 10 studies examining the associations between lipoproteins and IS, as well as its subtypes. The main characteristics of these studies are provided in Table 1. Most of the genetic data for the exposures were from individuals of European ancestry, with one study from Asian ancestry and another from African ancestry. The exposures of interest included LDL-C, HDL-C, triglycerides, Lp(a), apoA1, and apoB. All studies employed a two-sample MR approach, with IVW being the primary method for instrumental variable analysis. We assessed the quality of these MR studies using the STROBE-MR guidelines, and all were evaluated as high quality (Table 2).

**Table 1:** Study characteristics of all 10 studies included for qualitative analysis.

Study	Ethnicity	Exposure	Cohort	Outcome	Sample size	Design of MR study	Genetic instrument
Xia et al. (2021) (17)	East Asian	Lp(a)	CHCN-BTH	GWAS	Not Reported	Two-sample MR	13
Pan et al. (2019) (18)	European	Lp(a)	UKB	MEGASTROKE	40,585/406111	Two-sample MR	9
Martín-Campos et al. (2023) (11)	European	LDL	UKB	GIGASTROKE	1,234,808	Two-sample MR	44
		HDL					81
		apoA1					66
		apoB					51
		Triglyceride					61
Yuan et al. (2020) (19)	European	LDL	UKB	MEGASTROKE	60,341/454450	Two-sample MR	187
		HDL					469
		Triglyceride					390
		apoA1					396
		apoB					213
Hou et al. (2024) (20)	European	LDL	GWAS	GWAS	Not Reported	Two-sample MR	337
Liu et al. (2024) (21)	European	LDL	Integrative Epidemiology Unit database	European Bioinformatics Institute	34,217/406111	Two-sample MR	249
		HDL					529
		apoA1					294
		apoB					198
		Triglyceride					325
Allara et al. (2019) (12)	European	LDL	Global Lipids Genetics Consortium	UK Biobank	4602/367703	Two-sample MR	183
		HDL					183
		Triglyceride					183
Fatumo et al. (2021) (22)	African	LDL	African Partnership for Chronic Disease Research study, the UK Biobank, and the Million Veteran Program	Consortium of Minority Population Genome-Wide Association Studies of Stroke	3734/18317	Two-sample MR	71
		HDL					41
		Triglyceride					27
Wang et al. (2022) (23)	European	Lp(a)	European populations from Neale Lab	GWAS	Not Reported	Two-sample MR	16
Hindy et al. (2018) (24)	European	LDL	188577Global Lipids Genetics Consortium	National Institute of Neurological Disorders and Stroke−Stroke Genetics Network	16,851/32473	Two-sample MR	75
		HDL					85
		Triglyceride					51

**Table 2:** Quality Assessment tool conducted based on adherence to the Strengthening the Reporting of Mendelian Randomization Studies (STROBE-MR) Guidelines for all 10 studies included in qualitative analysis.

Study and year of publication	Title and abstract	Background	Objectives	Study design and data sources	Statistical methods: main analysis	Software and pre-registration	Descriptive data	Main results	Sensitivity and additional analysis	Key results	Limitations	Interpretation	Generalizability	MR core assumptions	Total Score(out of 14)	Score (%)
Xia et al. (2021) (17)	1	1	1	0.5	1	1	0.5	1	1	1	1	1	1	1	13	92.8
Pan et al. (2019) (18)	1	1	1	1	1	0.5	1	1	1	1	1	1	1	1	13.5	96.4
Martín-Campos et al. (2023) (11)	1	1	1	1	1	1	1	1	1	1	1	1	1	1	14	100
Yuan et al. (2020) (19)	1	1	1	1	1	1	1	1	1	1	1	1	1	1	14	100
Hou et al. (2024) (20)	1	1	1	0.5	1	0.5	1	1	1	1	1	1	1	1	13	92.8
Liu et al. (2024) (21)	1	1	1	1	1	1	1	1	1	1	1	1	1	1	14	100
Allara et al. (2019) (12)	1	1	1	1	1	1	0.5	1	0.5	1	1	1	1	0.5	12.5	89.2
Fatumo et al. (2021) (22)	1	1	1	1	1	1	1	1	1	1	1	1	1	1	14	100
Wang et al. (2022) (23)	1	1	1	0.5	1	1	0.5	1	1	1	1	1	1	1	13	92.8
Hindy et al. (2018) (24)	1	1	1	1	1	0.5	1	1	1	1	1	1	1	1	13.5	96.4

Each item is scored between 0 and 1 for each criterion to yield a total score. Upon conversion of the quality assessment score to a percentage, scores of <75%, 75–85% and >85% were considered to indicate high, medium and low risk of bias, respectively.

### 3.3 Meta-analysis of the causal relationship between non-HDL lipoproteins and IS

To assess the causal relationship between lipoproteins and IS, we categorized lipoproteins into non-HDL and HDL lipoproteins. Total cholesterol and triglycerides were also included in the analysis because these lipids are important fractions in composing lipoproteins.

**Figure 2:**
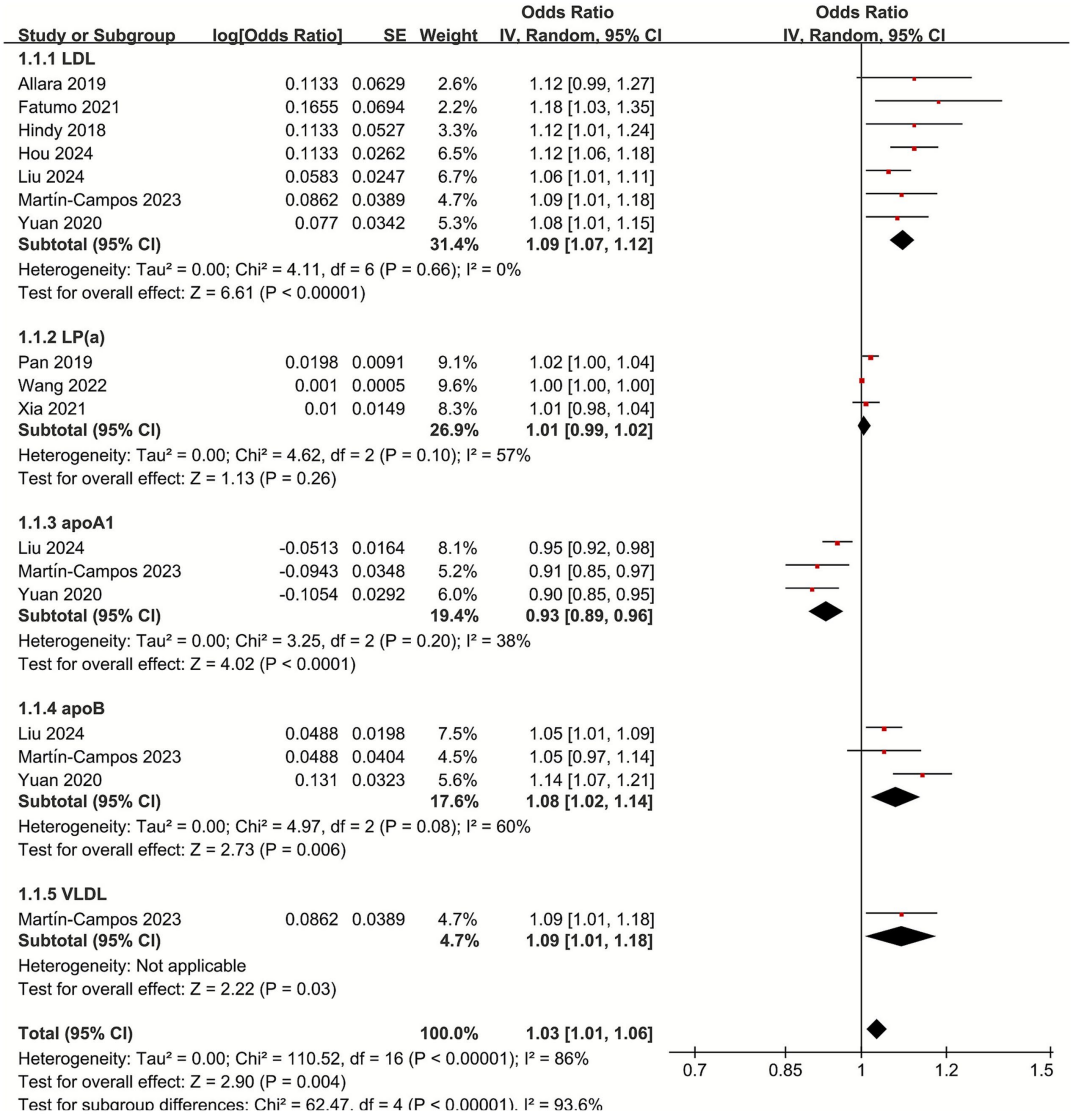
Forest plot of the non-HDL lipoproteins with subgroup analysis.

For non-HDL lipoproteins, the combined OR showed a significant positive correlation between LDL and IS (OR 1.09, 95% CI 1.07–1.12; *p* < 0.001, *I*^2^ = 0), and between apoB and IS (OR 1.08, 95% CI 1.02–1.14; *p* = 0.006, *I*^2^ = 60%). For VLDL, the only one MR study included in this meta-analysis showed a slight positive association between VLDL and IS (OR 1.09, 95% CI 1.01–1.18; *p* = 0.03), while the association between Lp(a) and IS was not statistically significant (OR 1.01, 95% CI 0.99–1.02; *p* = 0.26, *I*^2^ = 57%), there was a significant negative association between apoA1 and IS (OR 0.93, 95% CI 0.89–0.96; *p* < 0.001, *I*^2^ = 38%). Interestingly, although the association between Lp(a) and IS was not statistically significant, combined non-HDL lipoproteins were positively associated with IS (OR 1.03, 95% CI 1.01–1.06; *p* < 0.001, *I*^2^ = 93.6%) (Figure 2).

### 3.4 Meta-analysis of the causal relationship between HDL lipoproteins and IS

**Figure 3:**
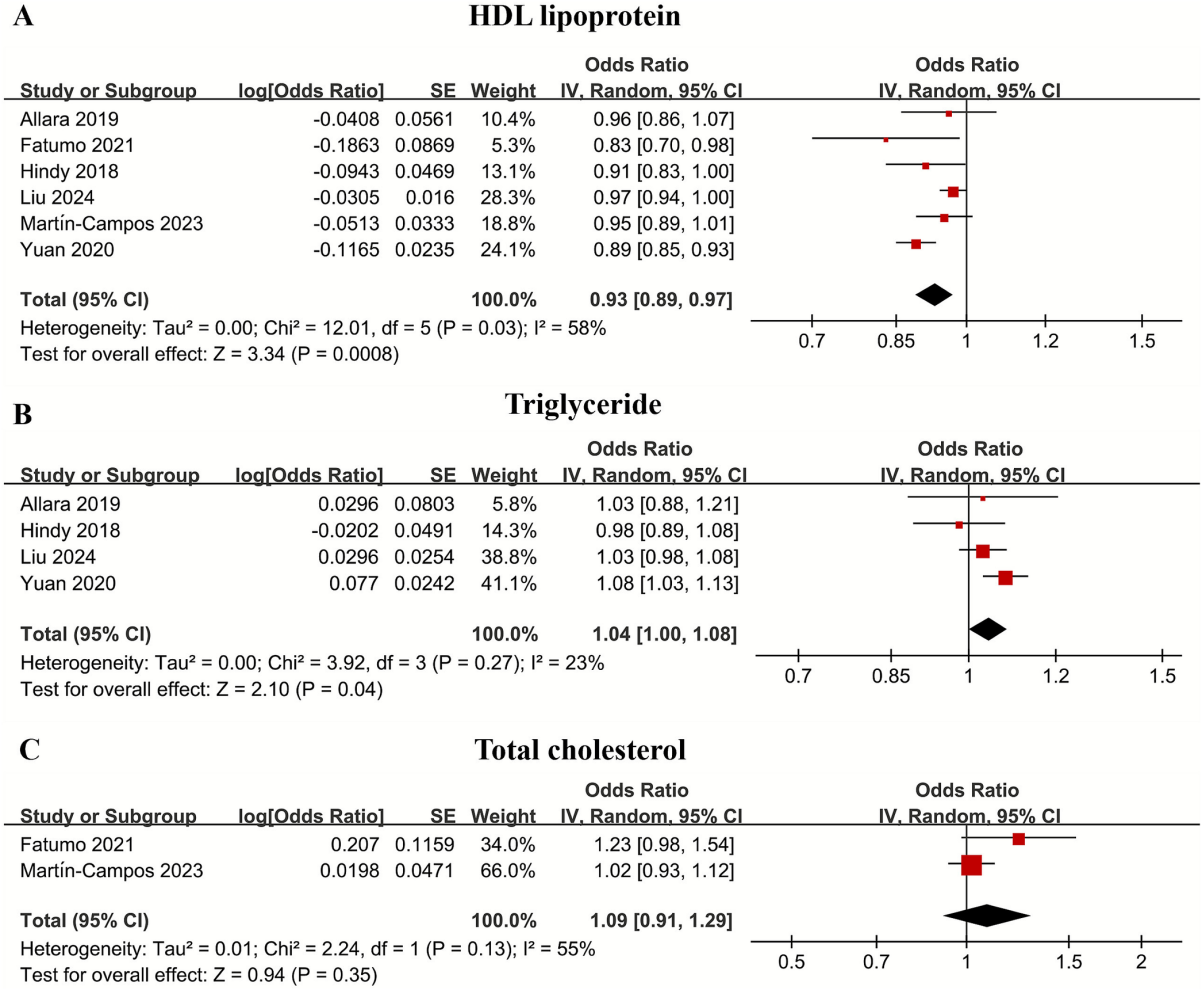
Forest plot of the HDL lipoproteins **(A)**, triglyceride **(B)**, and total cholesterol **(C)** with subgroup analysis.

For HDL lipoproteins, the pooled OR showed a significant negative correlation between HDL and IS (OR 0.93, 95% CI 0.89–0.97; *p* = 0.03, *I*^2^ = 58%). Triglycerides showed a slight positive correlation with IS (OR 1.04, 95% CI 1.00–1.08; *p* = 0.04, *I*^2^ = 23%) For total cholesterol, the pooled OR showed that the association between total cholesterol and IS was not statistically significant (OR 1.09, 95% CI 0.91–1.29; *p* = 0.35, *I*^2^ = 55%) (Figure 3).

### 3.5 Publication bias

**Figure 4:**
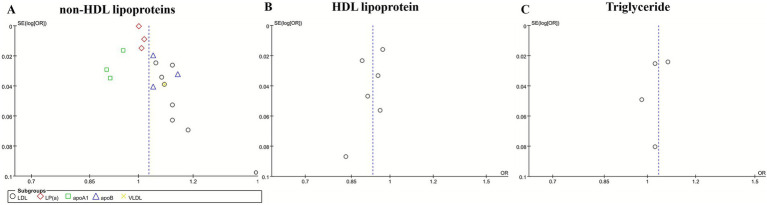
Funnel plot for non-HDL lipoproteins **(A)**, HDL lipoproteins **(B)**, and triglyceride **(C)**.

Publication bias among the included studies was assessed through the use of funnel plots. While the funnel plots demonstrated an approximately symmetrical distribution (Figure 4). The limited number of studies incorporated restricts the statistical power to identify potential asymmetry. Consequently, these findings should be interpreted with caution, and additional research is warranted to confirm these results.

### 3.6 Sensitivity analysis

To evaluate the robustness of causal associations between lipoprotein classes and IS, we conducted sensitivity analyses using a sequential exclusion approach for individual studies. The results revealed stable causal associations for LDL, apoA1, apoB, HDL, and total cholesterol with IS, whereas Lp(a) and triglycerides showed heterogeneity among the included studies.

## 4 Discussion

Through systematic evaluation and meta-analysis, this study aimed to identify which atherogenic lipid-related traits play a dominant role in contributing to IS risk. A total of 10 MR studies examining the causal relationships between various lipoproteins and IS were included in the analysis.

The pooled results of the meta-analysis demonstrated consistent evidence for causal associations between specific lipoproteins and IS. Most non-HDL lipoproteins—including LDL, apoB, and VLDL—were positively associated with an increased risk of IS. In contrast, HDL was causally associated with a reduced risk of IS. While apoA1 demonstrated a significant inverse association with IS, non-HDL lipoproteins exhibited a significant positive correlation with IS. Publication bias among the included studies was assessed through the use of funnel plots. While the funnel plots demonstrated an approximately symmetrical distribution (Figure 4), the limited number of studies incorporated restricts the statistical power to identify potential asymmetry. Consequently, these findings should be interpreted with caution, and additional research is warranted to confirm these results. The results of sensitivity analyses confirmed the robustness of the observed associations involving LDL, apoA1, apoB, HDL, and total cholesterol with IS.

Our analysis included only studies that focused on IS as the primary outcome and used the IVW method as the main analytical technique, which helped reduce clinical variability related to different stroke subtypes. Despite this, the combined non-HDL group still showed considerable heterogeneity (*I*^2^ = 93.6%). Moderate heterogeneity was also found for Lp(a), apoB, HDL lipoproteins, and total cholesterol. This variability may be due to differences in the number of SNPs selected in each study and the ethnic diversity of the populations studied. The overall pooled estimate might be concealing important causal effects within certain subgroups, which future research should aim to investigate. In contrast, LDL, apoA1, and triglycerides demonstrated low heterogeneity, indicating consistent effects across various genetic backgrounds and study designs. This suggests that the causal associations of these lipid traits with IS are relatively robust.

The results of our meta-analysis with the MR study are consistent with previous observational line studies that have identified a role for LDL-C, ApoB, and triglycerides in promoting the development of IS. Large observational studies (25) support the causal role of LDL as a cause of IS, and LDL has been identified as one of the major risk factors for IS. High LDL levels have also been recognized as independent risk factors for large-artery atherosclerotic and small-vessel occlusive stroke (26). The INTERSTROKE study (27) further confirmed that elevated LDL and ApoB are associated with an increased risk of IS, while HDL is linked to a reduced risk of IS. Additionally, apoA1 was found to be associated with a lower risk of IS. The protective effect of apoA1 is attributed to its crucial role in cholesterol metabolism and HDL synthesis, as well as its involvement in regulating inflammatory and immune responses associated with atherosclerosis, amyloidosis, and cardiovascular diseases (28). Observational studies have also found that higher ApoB levels correlate with an increased risk of IS (29). The study found that the proportion of IS associated with elevated apoB or non-HDL cholesterol was higher than that associated with elevated LDL cholesterol. This new finding suggests that elevated apoB and non-HDL cholesterol levels are a better indicator of IS risk than elevated LDL cholesterol levels (30).

Triglycerides are thought to be strongly associated with atherosclerosis. Observational studies have found that high triglyceride levels are significantly associated with an increased prevalence of intracranial arterial stenosis, but not extracranial arterial stenosis (31). Akhtar et al. (32) conducted a prospective cross-sectional study of 6,558 stroke patients, and in their study, they found that higher triglyceride levels were associated with IS, which is in line with our meta-analysis of MR study results. Statins, Ezetimibe, and the Proprotein Convertase Subtilisin/Kexin type 9 (PCSK9) Play an Important Role in the Prevention and Treatment of IS by Lowering LDL-C and Triglycerides (33).

Lp(a), a low-density lipoprotein cholesterol-like particle bound to apolipoprotein a, has been identified as an independent risk factor for atherosclerotic cardiovascular disease. Whether Lp(a) can be used as a potential biomarker for IS has received much attention, but there is still a lack of conclusive evidence for a causal relationship between Lp(a) and IS. Small-cohort prospective case–control studies have found (34) that Lp(a) may be an independent risk factor for IS, with fibrinogen acting as a partial mediator (35). The Reasons for Geographic and Racial Differences in Stroke (REGARD) cohort, which measured black and white Lp(a) levels in the general U. S. population, did not find an association between Lp(a) and incident IS in black or white participants (36). Panza et al. (37) highlighted that Lp(a) levels may vary by race and sex. An Atherosclerosis Risk in Communities (ARIC) study found that high Lp(a) concentrations were associated with a higher incidence of IS in white women, but not in white men (38). Our combined estimates showed no statistically significant association between Lp(a) and IS, with high heterogeneity likely attributable to racial and gender differences. Further meta-analyses are needed to confirm these findings.

The absence of a significant causal link between Lp(a) and IS in our meta-analysis presents a compelling scientific question. This finding may reflect a key limitation of the MR approach in this context: while it robustly assesses the lifelong atherogenic burden of Lp(a)-carried cholesterol, it may be less sensitive to its conditional, non-lipid effects. The unique structure of Lp(a), incorporating an LDL-like particle and the plasminogen-like apolipoprotein(a), endows it with dual pro-atherogenic and pro-thrombotic potential. It is therefore plausible that the stroke risk conferred by Lp(a) is not a simple function of its plasma concentration but is instead modulated by an individual’s inflammatory and coagulation status. This could mean that Lp(a) acts as a potent risk modifier or a “risk amplifier” in specific clinical scenarios, rather than a universal, independent driver of stroke. The ultimate test of this hypothesis now lies with a new class of targeted therapies. The development of potent Lp(a)-lowering agents, such as antisense oligonucleotides (pelacarsen) and small interfering RNAs (olpasiran), provides a direct experimental pathway. The results from their ongoing cardiovascular outcomes trials are eagerly anticipated, as they will not only clarify Lp(a)'s causal role but also determine whether reducing Lp(a) levels can mitigate the residual risk that persists despite optimal control of conventional lipids like LDL-C.

Our MR findings support a causal contribution of several lipid traits to IS. However, the pathways connecting lipoproteins to cerebrovascular injury extend beyond mere lipid accumulation and involve interrelated metabolic and hemodynamic processes. The association of LDL-C with IS is conventionally attributed to its role in atherosclerotic plaque formation. Yet, emerging evidence suggests that LDL-C also impairs endothelial function and arterial distensibility. Importantly, lowering LDL-C has been associated with improved vascular elasticity, as measured by pulse wave velocity, following therapeutic interventions such as inclisiran (39). This suggests that part of the benefit of LDL reduction may stem from restored vascular compliance. Similarly, triglyceride-rich lipoproteins, including VLDL, appear to promote cerebrovascular risk through both atherosclerotic and non-atherosclerotic mechanisms. Visceral adipose tissue-driven hypertriglyceridemia has been linked to increased arterial stiffness and residual cardiovascular risk, even among normoglycemic individuals (40). This pathway may help explain the causal relationship between VLDL and IS observed in our analysis, highlighting triglycerides as a mediator of early microvascular and macrovascular damage. Meanwhile, the protective association of HDL with IS likely involves its central role in reverse cholesterol transport, alongside anti-inflammatory and endothelial-stabilizing properties. By contrast, the non-significant association of Lp(a) in our meta-analysis may reflect heterogeneity in study populations or underlying differences in pathophysiology across stroke subtypes. Taken together, these observations suggest that lipoprotein-associated stroke risk represents the clinical endpoint of a dynamic metabolic–vascular remodeling process. This process encompasses not only luminal narrowing due to plaque, but also functional impairment of arterial tone and compliance. Further research is needed to dissect how specific lipid fractions interact with vascular biology across different stroke etiologies.

Despite these insights, our study has several limitations. The lack of individual patient-level data limited our ability to perform subgroup analyses based on race, age, and gender. Moreover, although MR provides valuable insights into causal relationships, it does not allow for precise quantification of effect sizes, which restricts its applicability in assessing the impact of interventions. Therefore, MR findings should be interpreted in conjunction with results from other epidemiological studies, rather than considered in isolation.

While this meta-analysis provides a systematic overview of the causal roles of lipoproteins in IS, several methodological aspects warrant careful consideration. The synthesis of MR studies introduces specific challenges that may contribute to the observed heterogeneity. First, the included studies employed different genetic instruments for the same exposure, varying in the number of instrumental single-nucleotide polymorphisms (SNPs), linkage disequilibrium clumping criteria, and statistical thresholds for SNP inclusion. These differences in instrument selection can influence the strength and precision of causal estimates. Second, variations in the source populations—including differences in ancestral genetic background and baseline risk factor distributions—may affect the transferability of genetic associations and causal estimates. Finally, although all studies examined IS, subtle differences in phenotypic definitions or case adjudication across consortia and biobanks could introduce further heterogeneity. While the random-effects model and sensitivity analyses helped account for some of this variability, these factors should be considered when interpreting the pooled results. Future studies would benefit from more standardized approaches to instrument selection and outcome harmonization.

## 5 Conclusion

This meta-analysis provides comprehensive evidence demonstrating the causal associations between various lipoprotein subclasses and IS. Elevated levels of non-HDL lipoproteins, particularly LDL and apoB, were shown to significantly increase the risk of IS, highlighting their key role in IS development. In contrast, higher concentrations of HDL and apoA1 were associated with a reduced risk of IS, indicating a protective effect and underscoring the importance of maintaining a healthy lipid profile for cerebrovascular well-being. While the association between Lp(a) and IS was not statistically significant, its potential impact cannot be excluded and warrants further investigation. Overall, these findings emphasize the vital role of lipid management in preventing IS and support continued efforts to better understand the mechanisms linking lipoprotein metabolism to cerebrovascular disease.

## Data Availability

The original contributions presented in the study are included in the article/supplementary material, further inquiries can be directed to the corresponding author.
